# Update on vascular control of central chemoreceptors

**DOI:** 10.1113/EP091329

**Published:** 2023-12-28

**Authors:** Thiago S. Moreira, Daniel K. Mulkey, Ana C. Takakura

**Affiliations:** ^1^ Department of Physiology and Biophysics, Instituto de Ciencias Biomedicas Universidade de Sao Paulo Sao Paulo Brazil; ^2^ Department of Physiology and Neurobiology University of Connecticut Storrs Connecticut USA; ^3^ Department of Pharmacology, Instituto de Ciencias Biomedicas Universidade de Sao Paulo São Paulo Brazil

**Keywords:** breathing, chemoreflex, Parkinson's disease, purinergic signalling, retrotrapezoid nucleus

## Abstract

At least four mechanisms have been proposed to elucidate how neurons in the retrotrapezoid (RTN) region sense changes in CO_2_/H^+^ to regulate breathing (i.e., function as respiratory chemosensors). These mechanisms include: (1) intrinsic neuronal sensitivity to H^+^ mediated by TASK‐2 and GPR4; (2) paracrine activation of RTN neurons by CO_2_‐responsive astrocytes (via a purinergic mechanism); (3) enhanced excitatory synaptic input or disinhibition; and (4) CO_2_‐induced vascular contraction. Although blood flow can influence tissue CO_2_/H^+^ levels, there is limited understanding of how control of vascular tone in central CO_2_ chemosensitive regions might contribute to respiratory output. In this review, we focus on recent evidence that CO_2_/H^+^‐induced purinergic‐dependent vasoconstriction in the ventral parafacial region near RTN neurons supports respiratory chemoreception. This mechanism appears to be unique to the ventral parafacial region and opposite to other brain regions, including medullary chemosensor regions, where CO_2_/H^+^ elicits vasodilatation. We speculate that this mechanism helps to maintain CO_2_/H^+^ levels in the vicinity of RTN neurons, thereby maintaining the drive to breathe. Important next steps include determining whether disruption of CO_2_/H^+^ vascular reactivity contributes to or can be targeted to improve breathing problems in disease states, such as Parkinson's disease.

## Abstract (highlights)

- **What is the topic of this review?**
  The homeostatic mechanism that controls breathing in response to changes in CO_2_/H^+^ is exquisitely sensitive, with convergent roles proposed for chemosensory brainstem neurons in the retrotrapezoid nucleus (RTN), their supporting glial cells and the vasculature.
- **What advances does it highlight?**
  We focus on recent evidence that CO_2_/H^+^‐induced purinergic‐dependent vasoconstriction in the RTN region supports respiratory chemoreception. This mechanism appears to be unique to the RTN and opposite to other medullary chemosensor regions, where CO_2_/H^+^ elicits vasodilatation. We speculate that this mechanism helps to maintain CO_2_/H^+^ levels in the vicinity of RTN neurons, thereby maintaining the drive to breathe.

## 1 INTRODUCTION

Breathing, the seemingly involuntary act that sustains life, is a complex and highly regulated process essential for maintaining adequate levels of O_2_ and CO_2_ in the bloodstream. Although we often take each breath for granted, the mechanisms underlying the control of respiration are intricate and finely tuned to ensure that the metabolic and physiological needs of the body are met (Del Negro et al., 2018). Central to this control is the CO_2_‐dependent drive to breathe, a fundamental aspect of respiratory physiology that governs the rate and depth of our inhalations and exhalations (Guyenet & Bayliss, 2015). Understanding the intricate interplay between CO_2_ levels and respiratory drive is not only essential for comprehending the basics of human physiology but also for unravelling the mysteries of numerous pathological conditions.

In addition, within this intricate orchestration of breathing regulation lies the vascular component of the CO_2_ drive to breathe, a less explored but important facet of the respiratory control system (Cleary et al., 2020; Hawkins et al., 2017; Oliveira et al., 2022). This component harnesses the power of the circulatory system to detect and respond to changes in blood CO_2_ levels, thereby influencing our breathing patterns. Moreover, the role of purinergic mechanisms within this context emerges as a fascinating and intricate piece of the puzzle, offering insights into the finer details of how our bodies regulate breathing in response to varying metabolic demands.

In this review, we describe the vascular component of the CO_2_ drive to breathe and the pivotal role played by purinergic mechanisms in both physiological conditions and neurological diseases, such as Parkinson's disease (PD). Parkinson's disease primarily affects the CNS and is often associated with classic motor symptoms (tremors, rigidity and bradykinesia) and other non‐motor symptoms (autonomic and respiratory deficits). Although the primary pathology of PD is related to the degeneration of dopamine‐producing neurons in the substantia nigra, it can also have secondary effects on various neurons that ultimately affect different physiological systems, including the respiratory system. Furthermore, we unravel the enigmatic system of purinergic signalling, a complex web of molecular messengers that play a vital role in modulating vascular responses and ultimately impact our breathing patterns.

Understanding the vascular component of the CO_2_ drive to breathe and the involvement of purinergic mechanisms not only provides us with insights into the subtleties of respiratory regulation but also has potential implications for various clinical scenarios. From respiratory disorders to cardiovascular diseases and neurological conditions, the knowledge gained from these mechanisms might shed light on the pathophysiological underpinnings of these disorders and open new avenues for therapeutic interventions.

## 2 THE MULTICELLULAR CHEMOSENSOR CONCEPT

The respiratory chemoreflex is a physiological reflex that can be triggered by the binding of a molecule to a receptor (Guyenet, 2014). Within the domain of respiratory research, the term ‘central chemoreception’ typically pertains to the process through which CO_2_, though the proxy of H^+^, activates respiratory control centres, primarily in the brainstem. However, a significant challenge arises from the fact that neither the specific ligand (such as molecular CO_2_, bicarbonate, protons or hydroxide ions) nor its corresponding receptors have been clearly identified. Additionally, the specific types of CNS cells that express these relevant receptors and trigger respiratory responses remain incompletely characterized (Guyenet et al., 2019; Moreira et al., 2021). Owing to these unresolved issues, the concept of central respiratory chemoreceptors remains a work in progress. In this context, we believe a better term to use should be CO_2_ sensors instead of CO_2_ chemoreceptors. This is because CO_2_ sensors are not only receptors located in neurons, but also different sensors that can be in glial cells, pericytes and smooth vascular cells that will contribute to the modulation of respiratory activity. Therefore, the challenge of confirming whether a specific cell type, such as neurons, astrocytes, pericytes or blood vessels, contains a genuine respiratory chemosensor is compounded by the concept of multicellularity. However, to establish that a cell of any type contains a legitimate respiratory chemosensor, some pieces of evidence are required. For example: (1) once activated or inhibited, the CO_2_ sensor must affect breathing output; (2) cell‐specific inhibition dampens the effects of CO_2_ on breathing; (3) in vivo or in vitro, CO_2_ sensor activity is modulated by CO_2_/H^+^ through a direct effect; and (4) specifically disrupting the cellular and molecular CO_2_ sensing mechanism within the CO_2_ sensor cells has an impact on the ventilatory response to hypercapnia. To date, no single candidate for the central respiratory chemosensor has satisfied all these criteria. However, we posit that retrotrapezoid nucleus (RTN) cells are on the verge of becoming an excellent candidate. These neurons exhibit robust responses to hypercapnia, both in vivo and in vitro (Mulkey et al., 2004; Takakura et al., 2006, 2013). Moreover, we possess a detailed understanding of their phenotype (Shi et al., 2017; Stornetta et al., 2006). The RTN has significant disease relevance, particularly in congenital central hypoventilation syndrome, and the cellular and molecular mechanisms involved in CO_2_ detection have been studied extensively in comparison to other CO_2_ chemosensors (Amiel et al., 2009; Ferreira et al., 2022; Kumar et al., 2015).

The phenomenon of increased respiration triggered by an increase in the partial pressure of CO_2_ in the brain, known as the hypercapnic ventilatory reflex, has been recognized for a considerable period (Loeschcke, 1982; Pappenheimer et al., 1965). As already mentioned, our understanding of the entities responsible for CO_2_ detection, including neurons, astrocytes and blood vessels, their precise locations within the CNS, and the molecular identities of CO_2_/H^+^ detectors remain incomplete. The predominant hypothesis suggests that CO_2_ detection occurs primarily in the ventral medullary surface, in a region with specialized neurons (retrotrapezoid chemosensor neurons, astrocytes and blood vessels) (Gourine et al., 2010; Hawkins et al., 2017; Kumar et al., 2015; Mulkey et al., 2004; Wenker et al., 2012). This region is called the respiratory parafacial region (Del Negro et al., 2018).

Several previous reviews have thoroughly covered the main chemosensory function of the ventral aspect of the respiratory region, termed the retrotrapezoid region (Guyenet, 2014; Guyenet & Bayliss, 2015; Guyenet et al., 2019; Nattie & Li, 2012). Instead, our review focuses on recent scientific discoveries pertaining to the vascular component of the CO_2_ drive to breathe.

Normally, an increase in CO_2_ levels in the brain (hypercapnia) leads to the dilatation of blood vessels. However, if this dilatation occurs in a region containing CO_2_ sensors, it can result in an increased blood flow that washes away locally generated CO_2_. This, in turn, diminishes the ability of chemosensitive neurons or glia to detect changes in arterial CO_2_ levels, as outlined by Xie et al. (2006). A unique feature within the RTN might counteract this potential effect of CO_2_. In contrast to the cortex or other brainstem respiratory centres, including the nucleus of the solitary tract (NTS) and medullary raphe, where acidification results in vasodilatation, arterioles in the RTN constrict in response to acidification (Cleary et al., 2020). Despite the opposite polarity, CO_2_/H^+^ vascular reactivity in both the cortex and the RTN can be blunted by blocking ATP‐purinergic receptors (Hawkins et al., 2017). At the level of the ventral parafacial region, astrocytes are thought to be the source of CO_2_/H^+^‐evoked ATP release. For example, expression of channel rodopsin (Ch2R) under the glial fibrillary acidic protein (GFAP) promotor (Gorine et al., 2010) or bath application of trans‐1‐aminocyclo‐pentane‐1,3‐dicarboxylic acid (tACPD) to elicit Ca^2+^ events in astrocytes (Hawkins et al., 2017) caused the activation of local neurons or triggered vasoconstriction by a purinergic‐dependent mechanism. If ATP originates from astrocytes, the narrowing of blood vessels and limited removal of CO_2_ could explain, in part, why the optogenetic depolarization of parafacial astrocytes stimulated breathing by activating RTN neurons and influencing breathing, as demonstrated by Gourine et al. (2010).

## 3 MECHANISMS UNDERLYING THE VASCULAR REGULATION OF BREATHING

The regulation of blood flow in the brain involves several intricate mechanisms, including neural activity, which induces vasodilatation and enhances blood flow through a process known as neurovascular coupling (Iadecola, 2017; Schaeffer & Iadecola, 2021). Autoregulation is another crucial mechanism that ensures the maintenance of cerebral blood flow when changes in perfusion pressure occur (Paulson et al., 1990). Notably, myogenic tone increases with elevated perfusion pressure and relaxes when pressure decreases. Additionally, CO_2_ and H^+^ ions serve as potent vasodilators in most brain regions (Hoiland et al., 2019). This regulatory role is particularly significant, because high levels of CO_2_ and H^+^ can impair both autoregulation (Ogoh & Ainslie, 2009; Perry et al., 2014) and neurovascular coupling (Maggio et al., 2013, 2014). Given that CO_2_ and H^+^ are metabolic waste products, their vascular reactivity offers a mechanism for aligning local blood flow with the metabolic demands of tissues. However, within the RTN region, this need seems secondary to the role of the chemosensor in regulating respiratory activity. For example, exposure to high levels of CO_2_/H^+^ has been shown to cause constriction of arterioles in the ventral parafacial region, which will conceivably help to maintain tissue H^+^ levels and the hypercapnic ventilatory response (Cleary et al., 2020; Hawkins et al., 2017; Wenzel et al., 2020). Consistent with this, application of a vasodilator or vasoconstrictor to the ventral medullary surface of anaesthetized rats decreased or increased CO_2_/H^+^‐dependent respiratory activity, respectively (Hawkins et al., 2017).

The underlying cellular and transmitter mechanisms responsible for the CO_2_/H^+^‐induced constriction of RTN vessels seem to involve the release of ATP from local astrocytes in response to CO_2_/H^+^. We know from the literature that CO_2_/H^+^ leads to discrete ATP release in the vicinity of the RTN region and that the activation of astrocytes induces RTN arteriolar constriction via a purinergic (P2Y_2_)‐dependent mechanism (Cleary et al., 2020; Hawkins et al., 2017). In addition, blockade of the P2Y_2_ receptors at the level of RTN vessels reduces the ventilatory response to CO_2_, demonstrating that the CO_2_/H^+^ vascular responses in the ventral parafacial region differ from other brain regions and are mediated by smooth muscle P2Y_2_ receptors (Cleary et al., 2020). The P2Y_2_ receptors are metabotropic receptors, and their activation in smooth muscle is associated with vasoconstriction (Brayden et al., 2013; Lewis et al., 2000). In contrast to the ventral parafacial region, increased levels of CO_2_ induce vessel dilatation in most other brain regions, which serves to couple blood flow with metabolic activity (Ainslie & Duffin, 2009). The sensitivity of the global brain vasculature to CO_2_ suggests an efficient mechanism for removal of excess CO_2_ and regulation of brain pH. However, a different mechanism was described for a brain region that contains cells that regulate breathing in response to increased levels of CO_2_. Increased levels of CO_2_ induce vasoconstriction in the ventral parafacial region, supporting the drive to breathe by maintaining tissue acidification and thereby stimulating chemosensors in this region.

Although it remains unverified, there is a reasonable hypothesis that vascular endothelial cells within the ventral parafacial region might release other vasoactive signals in response to CO_2_ to influence vascular tone. For example, exposure to high CO_2_ has been shown to facilitate prostaglandin E_2_ (PGE_2_) release in the parafacial region, which might contribute to RTN chemosensation directly or indirectly by modulation of vascular tone (Forsberg et al., 2016). Although this molecule is recognized for its roles in inflammation and fever, in the CO_2_ sensing region of the brainstem, it serves as a signalling molecule that heightens activity. Consequently, inflammation has the potential to disrupt the typical response of the body to CO_2_, leading to potentially life‐threatening respiratory issues. Additionally, PGE_2_‐induced deeper breaths, commonly known as sighs, might be crucial for newborns in taking their initial deep breaths. The forthcoming challenges involve unravelling the intricacies of how brainstem neural networks generate breathing and applying this understanding to enhance the treatment of respiratory difficulties in infants. Physiologically, we may hypothesize that elevated CO_2_ levels lead to Ca^2+^ oscillations in astrocytes, triggering increased arachidonic acid metabolism and the release of PGE_2_. This release of PGE_2_ might contribute to RTN chemosensation through a mechanism that involves EP3 receptors (Forsberg et al., 2016, 2017); however, given that The prostaglandin E receptor 3 (EP3) receptors are Gi coupled, we expect PGE_2_ signalling to inhibit the activity of RTN neurons. Nonetheless, this possibility is yet to be tested, and additional research is required to elucidate the physiological mechanisms of whether and how CO_2_ induces vasoconstriction and plays a role in respiratory control.

## 4 MECHANISMS UNDERLYING THE VASCULAR REGULATION OF BREATHING IN PARKINSON'S DISEASE

Parkinson's disease, a prevalent movement disorder characterized by a deficiency of dopamine and accumulation of the presynaptic protein α‐synuclein, affects ∼2%–4% of the population >60 years old around the world (Blandini, 2013). Besides the classic motor symptoms, PD patients can develop alterations in breathing output and in the ventilatory response to CO_2_ (Bernal‐Pacheco et al., 2012; D'Arrigo et al., 2020; Serebrovskaya et al., 1998).

As described above, hypercapnia elicits vasoconstriction in RTN vessels by a P2Y_2_‐dependent mechanism and is required for the normal ventilatory response to CO_2_ (Cleary et al., 2020; Hawkins et al., 2017). Recently, it was demonstrated that CO_2_/H^+^‐induced constriction was enhanced in a rodent model of PD (Oliveira et al., 2022). The increased vasoconstriction has the following potential explanations: (1) a higher number of purinergic receptors in the smooth muscles of RTN vessels; or (2) reduced activity of the ectonucleotidases. Given that ATP can convert rapidly into adenosine by enzymatic processes and considering the well‐established relationship between purinergic signalling, RTN astrocytes and blood vessels, CO_2_‐induced ATP released from astrocytes is metabolized into adenosine (James et al., 2018). This, in turn, might directly or indirectly modulate the activity of chemosensitive RTN neurons, possibly via an action in the smooth muscles of blood vessels in the RTN region (Cleary et al., 2020; Hawkins et al., 2017). Interestingly, the vasoconstriction induced by CO_2_ in RTN blood vessels was more prominent in a rodent model of PD when compared with control littermates (Oliveira et al., 2022). Based on multidimensional proteomic analysis, it was demonstrated that the regulation of ATP machinery in the region of the RTN is disrupted in the animal model of PD, affecting neurovascular coupling and, consequently, the regulation of breathing. These disrupted mechanisms heighten the sensitivity of RTN vascular smooth muscle cells and endothelial cells to the vasoconstrictor effects of ATP released by hypercapnia.

In summary, we believe that in PD, an initial elevation in catalytic purinergic activity occurs at the level of ventral parafacial region, leading to the accumulation of ATP. This accumulation, in turn, induces a more pronounced constriction of the vessels located in this region. Despite the disruption of the ATP machinery leading to ATP accumulation, the vasoconstrictive effect will not significantly influence the hypercapnic ventilatory response. This is because of the concurrent reduction in the number of neurons in key areas involved in breathing control in this disease (Fernandes‐Junior et al., 2018, Lima et al., 2018; Oliveira et al., 2018, 2017, 2019; Tuppy et al., 2015). Consequently, we have observed a decrease in breathing output, both in baseline conditions and when challenged with stimuli such as hypercapnia.

## 5 CONCLUSION

**FIGURE 1:**
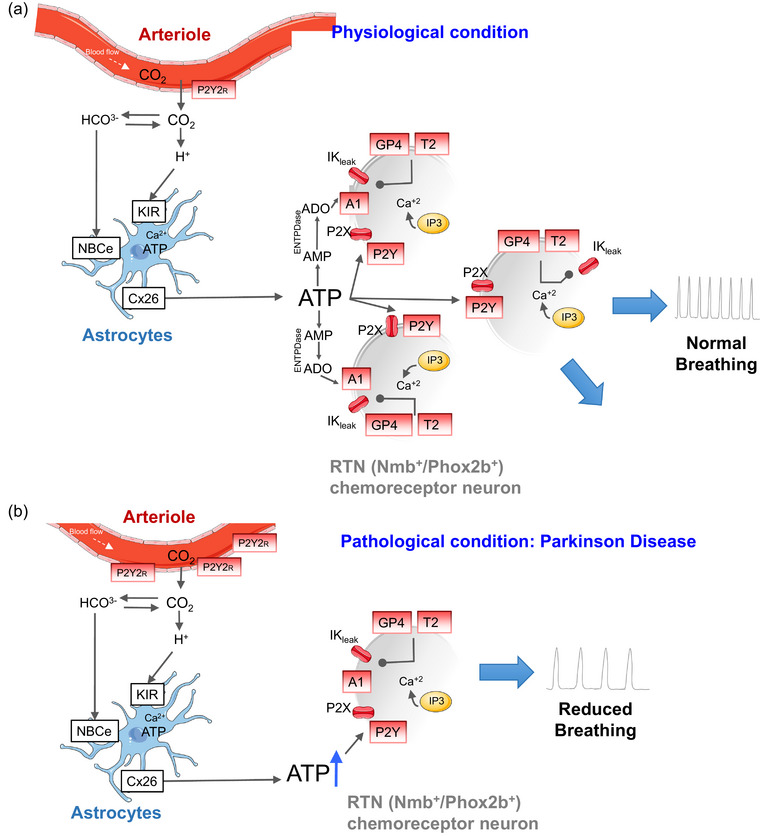
Possible interaction of neurons, astrocytes and blood vessels contributing to breathing regulation and chemosensitivity at the level of the respiratory parafacial region (from Oliveira et al., 2022; copyright by Elsevier). The activation of neurons of the ventral parafacial region, termed RTN neurons (Nmb^+^/Phox2b^+^), by CO_2_ requires the expression of two proton receptors (TASK‐2 and GPR4), but this response might be facilitated or potentiated by surrounding astrocytes and blood vessels. (a) In physiological conditions, extracellular acidification depolarizes RTN astrocytes by closing an inwardly rectifying potassium channel (KIR). This depolarization triggers the release of ATP (Gourine et al., 2010; Wenker et al., 2012), involving a calcium‐dependent exocytotic process triggered by intracellular acidification and/or a leak through connexin channels (primarily connexin 26) opened by molecular CO_2_ via carbamylation (Huckstepp et al., 2010). The ATP then contributes to the activation of RTN neurons via P2X and P2Y receptors, recruiting many astrocytes. Astrocyte depolarization might also activate an electrogenic sodium–bicarbonate transporter (NBCe), which moves bicarbonate into the cells, thereby further acidifying the extracellular space and enhancing the depolarization of RTN neurons (Erlichman et al., 2010). Recently, it was described that CO_2_ has the opposite effect on vascular tone in the RTN region, where the levels of CO_2_ are not only metabolic waste to be removed, but also function as a primary stimulus for breathing (Cleary et al., 2020; Hawkins et al., 2017). This mechanism apparently works through P2Y_2_ receptors at the level of vascular smooth muscle cells. (b) In pathological conditions, such as Parkinson's disease, significant biomolecular changes were observed in the RTN region, suggesting that the machinery of ATP was disrupted, affecting the interaction of neurons, glia and vessels and the control of breathing. Abbreviations: ADO, adenosine diphosphate; Cx26, connexin 26; ENTDase, ectonucleoside triphosphate diphosphohydrolase; GP4, G‐protein‐coupled receptor 4; IK_leak_, inwardly rectifying potassium channel‐bicarbonate; IP3, inositol triphosphate; KIR, inward‐rectifier potassium channels; NBCe, electrogenic sodium bicarbonate cotransporter; P2X, P2X purinergic receptor; P2Y2R, P2Y_2_ purinergic receptor; RTN, retrotrapezoid nucleus; T2, neuronal monocarboxylate transporter 2.

The region known as the ventral aspect respiratory parafacial region contains RTN neurons that, together with astrocytes and the surrounding vasculature, regulate breathing in response to CO_2_ (Guyenet et al., 2019; Moreira et al., 2021). Until now, several non‐exclusive mechanisms have been proposed to elucidate how RTN neurons respond to hypercapnia. These mechanisms include: (1) intrinsic neuronal sensitivity to H^+^ mediated by TASK‐2 and GPR4; (2) paracrine activation of RTN neurons by CO_2_‐responsive astrocytes (via purinergic mechanism); (3) synaptic inputs (excitatory inputs) and CO_2_‐induced vascular contraction (Figure 1). It remains uncertain whether the coexistence and interaction of these three mechanisms are unique to the RTN; however, it seems that CO_2_‐induced vasoconstriction at the level of the RTN is unique and has a physiological relevance by maintaining tissue acidification and thereby stimulating chemosensors in this region.

For more than a century, it was widely believed that an increase in CO_2_ levels led to the dilatation of all blood vessels in the brain, thereby enhancing blood flow. However, it was demonstrated recently that hypercapnia prompts blood vessels in the brainstem to constrict, whereas those in the rest of the brain dilate (Cleary et al., 2020; Hawkins et al., 2017). Although the pursuit of understanding central chemosensitivity has understandably focused on unravelling its cellular and molecular underpinnings, accumulating evidence underscores that central chemosensitivity emerges from intricate interactions among multiple cell types in the neurovascular coupling, with crucial physiological interactions at play. Although this phenomenon might seem relatively small in scale, its potential physiological implications should not be underestimated, especially in neurological diseases such as PD (Figure 1).

Further research is now imperative to address several key questions. Does the constriction of blood vessels observed at the RTN level influence the pH or the partial pressure of O_2_ of the surrounding tissue? Does this constriction impact the cellular physiology of neurons and astrocytes within the neurovascular unit? Addressing these questions could pave the way for a comprehensive understanding of the mechanisms underpinning central chemosensitivity at a systems level, offering insights into its variations in both health and disease.

## AUTHOR CONTRIBUTIONS

T.S.M., D.K.M. and A.C.T. wrote the paper and performed a critical review of the manuscript. All authors approved the final version.

## CONFLICT OF INTEREST

The authors declare no conflicts of interest.
